# A pilot study using environmental screening to determine the prevalence of *Mycobacterium avium* subspecies *paratuberculosis* (MAP) and antimicrobial resistance (AMR) in Irish cattle herds

**DOI:** 10.1186/s13620-020-0156-2

**Published:** 2020-02-15

**Authors:** Elvira Ramovic, Gillian Madigan, Shannon McDonnell, Denise Griffin, Elaine Bracken, Eadaoin NiGhallchoir, Emma Quinless, Aoife Galligan, John Egan, Deirdre M. Prendergast

**Affiliations:** Central Veterinary Research Laboratory, Department of Agriculture, Food and the Marine, Backweston Complex, Celbridge, Co. Kildare Ireland

**Keywords:** MAP, Antimicrobial resistance, *Salmonella*, ESBL, *E. coli*

## Abstract

**Background:**

Dairy and beef cattle can be reservoirs of many pathogens, including *Salmonella* and *Mycobacterium avium subsp. paratuberculosis* (MAP), the causative agent of Johne’s disease (JD). Farm environments may provide potential entry points for the transmission of infectious agents into the food chain. Antibiotics are used to treat a wide variety of infections on farms, and administration of antimicrobial agents to cattle is considered to be a driving factor for antimicrobial resistance (AMR). Control of JD and AMR are priority for animal health initiatives in Ireland. A national JD pilot programme was introduced by Animal Health Ireland in 2014, while the national action plan launched by Department of Health and Department of Agriculture, Food and Marine introduced in 2017 aims to improve the surveillance of AMR. The current investigation was undertaken as a pilot study to determine the proportion of herds positive for MAP, *Salmonella* species (*Salmonella* spp), commensal *Escherichia coli* (*E. coli)*, Extended-spectrum beta-lactamase (ESBL) AmpC β-lactamase and carbapenemase-producing *E. coli* from 157 environmental faecal samples in Irish farms.

**Results:**

MAP was detected in 10.2% of samples collected; on culture in 4 (4.9%) of the dairy herds and from 1 (1.3%) of the beef/suckler herds, and by PCR in 10 (12.3%) and 6 (7.9%) of these herds respectively. All culture positive herds were also positive by PCR. An additional 11 herds were positive by PCR only. *Salmonella* was not detected, while commensal *E. coli* were isolated from 70.7% of the samples (111/157) with 101 of these isolates shown to be fully susceptible to all antimicrobials tested. Of the 27 presumptive ESBL AmpC β-lactamase producing *E. coli* detected, one isolate was resistant to ten antimicrobials, nine isolates were resistant to nine antimicrobials, and four isolates were resistant to eight antimicrobials. Carbapenemase-producing *E. coli* were not isolated.

**Conclusions:**

The results highlight the importance of monitoring farm environments for Johne’s disease. This disease is a growing concern for dairy and beef producers in Ireland, and sampling the farm environment may offer a useful means to rapidly screen for the presence of MAP. Non-pathogenic common enteric commensal and multiple-drug-resistant *E. coli* may contribute to AMR acting as a reservoir and transferring resistance to other species/pathogens in the environment.

## Background

A wide variety of bacteria have natural reservoirs on the farm environment which may provide a potential entry point for transmission of infectious agents into the food chain. Therefore the occurrence of various pathogenic microorganisms on farms is of interest. Dairy cattle can be reservoirs of many pathogens, including *Salmonella*, a major foodborne zoonoses [1, 2] and *Mycobacterium avium subsp. paratuberculosis* (MAP), the causative agent of Johne’s disease (JD) [3]; a chronic intestinal disease in ruminants that may be associated with Crohn’s disease in humans [4]. In addition, other bacteria also present in cattle, which may or may not cause disease, and can act as reservoirs of antibiotic resistance which may also transfer to other bacteria or enter the food chain. *Escherichia coli* forms part of the normal microbiota of humans and animals and can spread through faecal material and wastewater in different environments [5]. Extended-spectrum beta-lactamase (ESBL) producing Enterobacteriaceae have emerged in the last decade as a global threat for human health [6]. They are not only isolated from hospital settings, but they are also disseminated in farm animals, their environments and animal-derived foods [7–11].

The use of antibiotics in veterinary medicine could constitute a selective pressure for the spread of antibiotic resistant bacteria including ESBLs [12]. Antibiotics are used to treat a wide variety of infections affecting animals on farms including respiratory and gastrointestinal diseases, reproductive disorders and mastitis in cows. Administration of antimicrobial agents to cattle is considered to be a driving factor for antimicrobial resistance (AMR) among *Salmonella* and other enteric pathogens [13–17].

Control of JD and antimicrobial resistance are now priority animal health initiatives in Ireland. A national JD pilot programme has been in operation since 2014 [18] and veterinary practitioners are encouraged to get client farms to participate and undertake herd sampling. Screening herds for antibodies to MAP is the primary method used to detect infection, but its low sensitivity and specificity can be challenging in some herds. Confirmatory testing using culture or PCR is advised to confirm infection in antibody positive animals.

A national action plan aimed at tackling the serious and increasing threat posed by AMR in Ireland was launched jointly by the Department of Health (DH) and Department of Agriculture, Food and the Marine (DAFM) in 2017. Among its five strategic objectives was the need to improve surveillance of AMR. Currently, the AMR surveillance undertaken in Ireland in food and animals is that required under Commission Implementing Decision on the monitoring and reporting of antimicrobial resistance in zoonotic and commensal bacteria [19] and obligatory monitoring focuses on resistance in *Salmonella*, *Campylobacter* and *E. coli* isolated from poultry, pigs and their meat. Currently very little attention is given to AMR on isolates from bovines although diagnostic laboratories in Ireland provide some data on clinical isolates to assist veterinary treatment of infections in these animals.

The current investigation was undertaken as a pilot study to determine the proportion of herds positive for MAP, *Salmonella* spp., commensal *E. coli*, ESBL AmpC - β lactamase and carbapenemase-producing *E. coli* on Irish farms from environmental faecal samples. The AMR profile in any *Salmonella* and *E. coli* isolates as well as the extent of ESBL AmpC β-lactamase or carbapenemase-producing *E. coli* was also determined.

## Methods

### Samples

Veterinary inspectors in each district office were requested to submit two farm faecal environmental samples, comprised of pooled environmental faeces collected from indoor locations (collecting yards, feeding areas, etc.), from both dairy and beef herds during their inspection visits in Ireland between February and June 2017.

Kits, including disposable gloves and sterile containers, were supplied for sample collection. Once the samples were collected they were submitted immediately by post to the laboratory for testing. Identification of herds was not requested nor was the information on any recent drug treatments, or details on the health status of herds. Once received at the laboratory, sample details (herd type, date and place of sample collection etc.) were recorded and samples were stored at − 20 °C for a maximum of 6 months before culturing. Prior to testing, the samples were removed from storage at -20 °C and defrosted overnight (approx. 18 h) at room temperature. The samples were cultured for MAP, *Salmonella*, commensal *E. coli* and also examined for the presence of ESBL AmpC β-lactamase and carbapenemase-producing *E. coli*.

### Statistical analysis

Test agreement between culture positive and PCR positive samples as well as Kappa value was calculated using https://epitools.ausvet.com.au/ [20]. The strength of agreement was interpreted as follows: value less than 20 was interpreted as poor agreement; values between 0.21–0.40 were fair; 0.41–0.60 were moderate; 0.61–0.80 indicated a good agreement, while values above > 0.81 were described as very good test agreement [21].

### MAP culture

MAP culture was performed using the Cornell double incubation decontamination method as previously described by Kim et al. (2002) [22]. In brief, after suspending faeces in 35 ml sterile water, the 5 ml from the top portion of the sample was transferred to 25 ml of 0.9% hexadexcylpiridinium chloride (HPC) (Sigma Aldrich) and incubated for 18–24 h at 37 °C, followed by an additional 18–24 h incubation in antibiotic brew (Trek diagnostic systems, Thermo Scientific, US). Samples were then inoculated into Trek bottles with added growth supplements for incubation at 37 °C for 42 days of continuous monitoring by the Versa Trek system (Trek diagnostic systems, Thermo Scientific, US). Positive and negative Versa Trek signal samples were subjected to Ziehl-Neelsen (ZN) staining, and samples that contained acid fast bacilli (AFB) and were further confirmed for MAP using mycobactin dependence [23] and F57 PCR [24] assays. MAP levels in samples were determined as described by Lombard et al. (2006) [25] using the days to positivity on the Versa Trek system. Samples signaling positive in the first 21 days of incubation indicated high MAP levels present, between 22 and 28 days indicated moderate MAP levels present, signals between 29 and 35 and 36–42 days indicated the presence of low and very low levels, respectively.

### Direct MAP PCR

Direct faecal PCR was carried out using the spin column (Qiagen DNA mini kit, Qiagen Ltd., Manchester, UK) extraction method [26] and LSI VetMAX M. *paratuberculosis* Advanced Real Time PCR kit (Thermo Fisher Scientific) targeting the *IS*900 sequence, following manufacturer’s extraction and purification recommendations. In brief, 2 g of faecal sample was suspended in 30 mL of sterile DNA/RNA free water and 3.6 mL from the top layer was used to obtain pellet. The buffer was added to the pellet and the sample was disrupted using the tissue lyser (Qiagen). Tissue lysis buffer and internal positive control (IPC) was further added to the sample and DNA extraction was carried out using the buffers and silica gel membrane spin column as described in part by Sting et al. (2014) [27]. In brief, ethanol and buffers (Qiagen) were added to the sample, and MAP DNA was eluted from spin column using elution buffer (Qiagen). Master mix containing buffer, real time PCR enzymes, sequence pool (LSI VetMAX) and 5 μl of eluted DNA was prepared and samples were analyzed using the Stratagene MxPro3005 thermo cycler, and a Ct value of less than 45 cycles was considered positive for MAP as recommended by the kit manufacturer.

Individual controls were included for each test to ensure validity of the test results and compliance with the accredited standards used in the testing laboratory.

### Isolation of commensal *E. coli*

Using a sterile cotton swab, the faecal sample was mixed thoroughly against the side of the sample container. The swab was streaked directly onto MacConkey (MAC) agar No. 3, E&O Laboratories Ltd., UK [Cat #: PP1720]) and then triple streaked using a sterile loop to isolate single colonies [28, 29]. The plates were incubated at 37 °C ± 1 °C for 20 ± 2 h and examined for typical *E. coli* growth (purple/red colonies). Typical colonies were sub-cultured onto Brilliance™ *E. coli*/Coliform (BECC) medium (Fannin L.I.P, Ireland [Cat#: W11106]), incubated at 37 ± 1 °C for 20 ± 2 h and examined for typical *E. coli* growth (purple colonies).

### Pre-enrichment of samples

A pre-enrichment step was performed by weighing 25 ± 1 g into a stomacher bag using a Dilumat (AES International). The sample was diluted 1 in 10 with Buffered Peptone Water (BPW) ISO (LAB M Ltd., Lancashire, UK), homogenised by stomaching for 1 min (360 cycles/min; speed setting 6) (Interscience BagMixer® 400 P) and incubated at 37 ± 1 °C for 18 ± 2 h. The pre-enriched samples were examined for the presence of ESBL AmpC β-lactamase and carbapenemase-producing *E. coli* and *Salmonella* following the laboratory protocol described by the EU Reference Laboratory for antimicrobial resistance [30].

### Isolation of ESBL/AmpC producing *E. coli*

Following overnight pre-enrichment, samples were gently mixed by hand and a 10 μl loopful of the enriched BPW was inoculated onto a MAC plate containing 1 mg L^− 1^ cefotaxime (CTX) (E&O Laboratories, Cat #: PP0478). The plates were incubated at 44 ± 0.5 °C for 20 ± 2 h and examined for typical *E. coli* growth as described above. Presumptive ESBL AmpC β-lactamase producing *E. coli* colonies were sub-cultured onto MAC agar plates containing 1 mg L^− 1^ CTX and incubated at 37 ± 1 °C for 20 ± 2 h. For species identification, suspect colonies were sub-cultured onto BECC plates, incubated at 37 ± 1 °C for 20 ± 2 h and examined for typical growth.

### Isolation of carbapenemase-producing *E. coli*

For isolation of carbapenemase-producing *E. coli* including strains producing only OXA-48 like enzymes, pre-enriched samples were gently mixed as above and a 10 μl loopful of the BPW was streaked onto onto a chromID™ CARBA plate (Biomérieux, Cat #: 43861) and chromID™ OXA-48 plate (Biomérieux, Cat #: 4414011) and incubated at 44 ± 1 °C for 20 ± 2 h. The plates were examined for typical *E. coli* growth (mauve colonies on both plate types). Presumptive colonies were subcultured onto MAC plates followed by BECC plates and examined for typical growth as described above for isolation of ESBL AmpC β-lactamase producing *E. coli*.

### *Salmonella* isolation

*Salmonella* were isolated following the standard protocol [31]. In brief, 100 ± 5 μl of the pre-enriched BPW was inoculated onto Modified semi-solid Rappaport Vassiliadis (MSRV) medium by placing 3 equidistant drops onto the surface of the agar in the central area of the plate. The inoculated MSRV plates were incubated at 41.5 ± 1 °C for 24/48 ± 3 h. Following 24 h incubation, or 48 h if no growth was observed at 24 h, presumptive *Salmonella* positive growth (turbid zone characterised by a white halo with a clearly defined edge) was subcultured onto Xylose Lysine Deoxycholate (XLD) (E&O Laboratories) and Modified Brilliant Green (MBG) selective agar plates (E&O Laboratories, Cat #: PP0320 and PP0060, respectively). The selective agar was incubated at 37 ± 1 °C for 24 ± 3 h and checked for typical *Salmonella* growth (black colonies on XLD agar and pink/red colonies on MBG agar). Any suspect colonies were sub-cultured onto Colorex *Salmonella* Plus (CHROMagar) (E&O Laboratories, Cat #: PP1071) plates, incubated at 37 ± 1 °C for 24 ± 3 h and examined for typical mauve coloured colonies for *Salmonella* growth.

### Species identification of *E. coli* and *Salmonella*

Presumptive *E. coli*, ESBL AmpC β-lactamase, carbapenemase-producing *E. coli* and *Salmonella* colonies were inoculated onto Nutrient Agar (NA; E&O Laboratories, Cat #: PP0690) and incubated at 37 ± 1 °C for 20 ± 2 h.

The MALDI Biotyper was used to identify microorganisms using MALDI-ToF (Matrix Assisted Laser Desorption Ionization-Time of Flight) Mass Spectrometry (Bruker Daltronics GmbH, Bremen, Germany). An individual colony from a NA plate was spotted onto the ground steel target plate (MSP 96 target polished steel reusable slides with barcode) of the MALDI-ToF. The entire spot was then overlain with 1 μl of pre prepared α-Cyano-4-hydroxycinnamic acid (HCCA) matrix solution and allowed to air dry. The matrix solution was prepared by adding 250 μl standard solvent (50% acetonitrile, 47.5% HPLC Water, 2.5% Trifluoroacetic acid) to the contents of one tube of Bruker HCCA Matrix to give a final HCCA concentration of 10 mg mL^− 1^. The solution was vortexed for approximately 10 s at room temperature followed by a 10 s spin in a mini centrifuge. This solution was stored at room temperature for up to 1 week.

Once the matrix was dry, the target plate was loaded onto the MALDI-ToF and measurements were performed with microflex mass spectrometer of the MALDI-ToF instrument using Compass software. The results were collected electronically in spectral channels and converted from TOF measurements into mass/charge values. A value ranging from 2.00 to 3.00 was interpreted as a highly probable species level identification [32].

In addition to the test samples, a bacterial test standard (BTS) quality control sample was included with each run for instrument calibration to ensure reliable and accurate identification of microorganisms. Pre-prepared BTS was added to the slides and overlaid with matrix as described above for each of the samples. The BTS was prepared by removing one tube of BTS (Bruker) from storage at − 18 °C, equilibrated to room temperature and 50 μl of standard solvent was added. BTS was dissolved by pipetting up and down at least 20 times. The standard was centrifuged at 13,000 RPM for 2 min at room temperature. Aliquots (5 μl) of the supernatant were pipetted into 0.5 mL screw-cap micro tubes and stored at − 18 °C. Frozen, dissolved BTS were stored for up to 5 months at − 18 °C.

All isolates confirmed by MALDI-ToF were stored at − 80 °C in Protect beads (Technical Service Consultants Ltd., Lancashire, U.K) prior to testing for AMR.

### AMR susceptibility testing of commensal *E. coli*

AMR testing was carried out using broth microdilution according to the regulation [19] and the results recorded as the Minimum Inhibitor Concentration (MIC) which is the lowest concentration of antimicrobial without visible growth. Beads were grown on Columbia Agar Base with 5% Defibrinated Horse Blood (E&O Laboratories, Cat #: PP0120) at 37 ± 1 °C for 20 ± 2 h and examined for pure growth. A 0.5 McFarland culture solution (1.5 × 10^8^ CFU/mL) [33] was prepared by suspending several colonies in demineralised water (Sensititre, Cat #: T3339) and adjusting the turbidity using a nephelometer (Sensititre). The 0.5 McFarland culture suspension was diluted by adding 10 μl of the suspension to a tube of Cation-adjusted Mueller-Hinton broth (MH) broth (Sensititre, Cat #: T3462) and mixed by inversion. Using an autoinoculator (ThermoFisher), the solution was inoculated onto EUVSEC Sensititre broth microdilution antimicrobial susceptibility plates (TREK Diagnostic Systems), 50 μl per well. The plates were sealed and incubated at 37 ± 1 °C for 20 ± 2 h. The following antibiotics were tested: Amp (ampicillin), Azi (azitromycin), Ctx (cefotaxime), Caz (ceftazidime), Chl (chloramphenicol), Cip (ciprofloxacin), Col (colistin), Gen (gentamicin), Mer (meropenem), Nal (nalidixic acid), Smx (sulphamethoxazole), Tet (tetracycline) and Tmp (trimethoprim). A post-sensitivity purity check was carried out by subculturing 1 μl of culture from the positive control well on the AMR plate onto a BA plate and incubated at 37.0 ± 1 °C for 20 ± 2 h. The AMR plate was read visually using an automatic Vizion plate reader (ThermoFisher) to determine the MIC. A control strain of *E. coli* 25,922 was tested alongside each batch of samples. The interpretation of the results, susceptible or resistant to a given antimicrobial, was carried out based on the EU decision [19].

### AMR susceptibility testing of *E. coli*, ESBL AmpC β-lactamase and carbapenemase-producing *E. coli*

AMR testing on presumptive *E. coli*, ESBL AmpC β-lactamase and carbapenemase-producing *E. coli* was performed using the two-step approach, i.e. both testing panels (EUVSEC and EUVSEC2). From the first panel (EUVSEC), *E. coli* strains which exhibited resistance to cefotaxime, ceftazidime and/or meropenem were further tested using the ESBL microtitre plate EUVSEC2.

The procedure that was followed was identical to the method outlined above for EUVSEC plates. The following antibiotics were tested: Fep (cefepime), Ctx (cefotaxime), CTX-C (cefotaxime/clavulanic acid), Fox (cefoxitin), Caz (ceftazidime), CAZ-C (ceftazidime/clavulanic acid), Etp (ertapenem), Imi (imipenem), Mer (meropenem) and Tem (temocillin). This second plate was used as a confirmatory test for ESBL production and required the use of both cefotaxime and ceftazidime alone or in combination with a β-lactamase inhibitor (Clavulanic acid). Synergy was defined as a ≥ 3 dilution decrease in MIC tested (in combination with clavulanic acid vs alone). The classification of the phenotypic results was based on the most recent EFSA recommendations [34].

## Results

Environmental faecal samples were collected from a total of 157 farms; 81 of which were dairy herds and 76 beef/suckler herds. The farms were distributed in 24 counties throughout Ireland; 43.1% in Munster, 19.6% in Leinster, and 15.7 and 21.6% in Connacht and Ulster respectively. No information of geographical location was obtained for 4 farms.

MAP was detected on culture in 4 (4.9%) of the dairy herds and from 1 (1.3%) of the beef/suckler herds and by PCR in 10 (12.3%) and 6 (7.9%) of these herds respectively. Kappa value between culture and PCR was calculated at 0.45 (95%CI 0.19 to 0.71) with overall proportion agreement calculated at 0.93. All the culture positive herds were also positive on PCR (Table 1).

**Table 1:** Environmental screening results of dairy and beef herds using culture and direct PCR assays

Province	Herd Type Dairy / Beef	Number of positive herds by Culture / PCR	
		Dairy^a^	Beef^b^
Munster	37/29	2/6	0/3
Leinster	18/12	0/0	0/1
Connacht	9/15	0/1	0/1
Ulster	14/19	2/3	0/0
Total^c^	78/75	4/10	0/5

^a^Three herds were recorded negative on both culture and PCR

^b^One herd was recorded positive on both culture and PCR

^c^Additional four herds were screened with unknown geographical location

Of the 5 culture positive samples, one was recorded as containing a high level of MAP, one a moderate level, and three samples as having very low levels of MAP present. Mean Ct value for culture positive samples was recorded at 31.75 (range: 29.43–33.46), while the mean Ct value for culture negative but PCR positive samples was 35.18 (range: 33.14–37.08).

*Salmonella* spp. was not isolated from any of the environmental samples. Commensal *E. coli* were isolated from 111 of the 157 samples. Following AMR, 101 of these isolates were shown to be fully susceptible to all antimicrobials tested (Table 2). Five were resistant to four antimicrobials with resistant patterns of Amp, Chl, Smx, Tet in four isolates and Amp, Chl, Cip, Nal in one isolate. Four isolates were resistant to three antimicrobials displaying resistant profiles of Amp, Smx, Tet and one isolate was resistant to two antimicrobials showing a resistant profile of Smx, Tet (Table 2).

**Table 2:** AMR profile of 111 commensal *E. coli* isolated from farm environmental samples

AMR Profile	Number of Isolates
Fully susceptible	101
Amp^a^, Chl^b^, Smx^c^, Tet^d^	4
Amp, Smx, Tet	4
Amp, Chl, Cip^e^, Nal^f^	1
Smx, Tet	1

^a^Ampicillin

^b^Chloramphenicol

^c^Sulphamethoxazole

^d^Tetracycline

^e^Ciprofloxacin

^f^Nalidixic acid

Following screening for the presence of ESBL, AmpC and carbapenemase-producing *E. coli*, 27 presumptive ESBL AmpC β-lactamase producing *E. coli* were isolated. No Carbapenemase *E. coli* were isolated. AMR on EUVSEC plates displayed 14 different AMR profiles for the 27 isolates (Table 3). One isolate was resistant to ten different antimicrobials (Amp, Ctx, Caz, Chl, Cip, Gen, Nal, Smx, Tet, Tmp), nine isolates were resistant to nine antimicrobials displaying four different AMR profiles and four isolates were resistant to eight antimicrobials displaying three different AMR profiles (Table 3).

**Table 3:** AMR profile of 27 presumptive ESBL AmpC β-lactamase *E. coli* isolated from farm environmental samples

AMR profile EUVSEC	EUVSEC 2	Final Interpretation^a^	Number of Isolates
Amp Ctx Caz Cip Nal Smx Tet Tmp	Fep Ctx Caz	Presumptive ESBL	1
Amp Ctx Caz	Fep Ctx Fox Caz	Presumptive ESBL	1
Amp Ctx Caz Chl Cip Nal Smx Tet Tmp	Fep Ctx Caz	Presumptive ESBL	3
Amp Ctx Caz Cip Nal Smx Tet Tmp	Fep Ctx Caz	Presumptive ESBL	1
Amp Ctx Caz Chl Cip Nal Smx Tmp	Fep Ctx Caz	Presumptive ESBL	2
Amp Ctx Caz	Ctx Caz	Presumptive ESBL	1
Amp Ctx Caz	Fep Ctx Fox Caz Etp	Presumptive ESBL + pAmpC	1
Amp Ctx Caz	Fep Ctx Fox Caz	Presumptive ESBL + pAmpC	1
Amp Ctx Caz Chl Cip Nal Smx Tet Tmp	Fep Ctx Fox Caz	Presumptive ESBL + pAmpC	1
Amp Ctx Caz	Ctx Fox Caz	Presumptive pAmpC	1
Amp Ctx Caz Chl Cip Gen Nal Smx Tet Tmp	Ctx Fox Caz	Presumptive pAmpC	1
Amp Ctx Caz Chl Cip Gen Nal Smx Tmp	Ctx Fox Caz	Presumptive pAmpC	2
Amp Ctx Caz Chl Cip Nal Smx Tet Tmp	Ctx Fox Caz	Presumptive pAmpC	3
Amp Ctx Caz Smx Tet	Ctx Fox Caz	Presumptive pAmpC	8

^a^Interpretation based on EFSA recommendations

All 27 presumptive ESBL AmpC β-lactamase isolates on EUVSEC plates were resistant to either Cefotaxime, Ceftazidime or both, and therefore EUVSEC2 plates were applied to these isolates. The results on EUVSEC2 plates permitted the final interpretation of ESBL Phenotype/Presumptive ESBL producer (nine isolates), Presumptive ESBL + pAMPC producer (3 isolates) and presumptive pAmpC phenotype/Presumptive AmpC producer (15 isolates) as shown in Table 3.

## Discussion

The farm is a dynamic environment and represents a possible entry point for pathogens directly into the food chain and indirectly through their dissemination into the agro-ecosystem through land-spreading of manures as nutrient sources for growing crops [35]. Once spread with manure to agricultural land, pathogens can survive for extended periods [3, 36–39] leading to the opportunity for contamination of food production and water supply systems [40, 41]. Cattle can be reservoirs for several pathogens, including *Salmonella* [1, 2] and MAP, the causative organism for Johne’s disease [3] a chronic intestinal disease in ruminants that may be associated with Crohn’s disease in humans [4]. Sampling the farm environment can therefore be a useful and convenient way [42–44] of screening for the presence of various pathogens, not unlike the analysis of boot swabs collected from the poultry farm environment which are routinely used for monitoring the presence of notifiable *Salmonella* [45].

Johne’s disease is a growing concern for dairy and beef producers in Ireland and elsewhere and programmes for its control are in place in many countries. MAP, the etiologic agent of Johne’s disease is a growing concern in Irish cattle herds and has been considered by some to be a potential emerging foodborne pathogen [35, 46–52]. Estimates of its prevalence in many countries vary and diagnostic tests have limited sensitivity, particularly for detecting early stages of infection [53]. Serological surveys estimate herd prevalence in dairy herds at 80–86% in Denmark, 65% in the UK and between 20 and 71% in Netherlands [54]. In Ireland [55], calculated seroprevalence at 21.4% with dairy herds having a higher incidence (31.5%) than beef herds (17.9%) while in a more recent study McAloon et al (2016) [56] using Bayesian analysis, calculated the true herd prevalence in the region of 23–34%. The current study is the first here in Ireland to use environmental sampling to estimate the extent of MAP on farms. Results of this study showed that MAP was detected in 16/157 farms and confirmed on culture in 4 (4.9%) dairy herds and 1 (1.3%) of the beef/suckler herds. Other Irish researchers have screened milk sock filter residue (MRF) [57] to estimate MAP prevalence on dairy farms and found 44% [26] compared to 20% MFR culture positive herds. In that study, among the 12 MFR culture positive herds, each was positive at only one of the six testing events over the two-year period.

A number of diagnostic tests are available for the detection of MAP, each with positive and negative attributes. Serological testing of animals is the primary screening method applied in Ireland for the detection of infected herds with follow up confirmatory testing of faeces from suspect animals by culture or PCR. Concerns about the ELISA test are reported as climate and cattle management systems on some Irish farms predispose animals through exposure to environmental mycobacteria and it is recognised that such exposure may give rise to non-specific or false positive MAP ELISA reactions [41, 58]. The Irish climate and abundant rainfall allow up to 10 months of pasture production per year; pasture based rotational systems are the norm on Irish dairy farms [59, 60]. Cross reaction to MAP may also result from administration of tuberculin [61, 62] which is used in the bovine tuberculosis control programme in Ireland [63] contributing to non-specific or false positive MAP ELISA reactions. In view of these concerns it might be opportune to further examine environmental sampling for MAP as an alternative screening method.

Cultivation of MAP, although expensive and slow still remains the “gold standard” diagnostic test for the disease [64] with an ante-mortem specificity of 100% [43, 65] and a sensitivity varying from 30 to 50% [25, 66–68]. Sensitivity of culture on a herd level is affected by the number of shedders present and their shedding levels [69, 70]. Seasonal influences may also affect culture in so far as increased fungal growth in faeces during the warmer season may not be fully removed during the decontamination process used in the culture method resulting in culture negative samples [3, 69, 71]. PCR is emerging as a comparable method to culture and can be completed in a day compared to up to 42 days or longer required for the culture results [26, 72]. Sensitivity of PCR is reported to be between 70 and 100%, depending on infection stage and pre-treatment methods applied in DNA extraction [65, 73], with specificity considered to be 100% [27, 73]. The inclusion of physical or chemical steps to improve DNA extraction, as well as the use of alternative MAP targets [74] have been shown to improve the sensitivity of the kits [75, 76]. While the *IS*900 sequence is considered highly sensitive and specific for MAP, a positive signal has also been reported from environmental mycobacteria [22, 77]. , Eisenberg et al. (2010, 2012) [78–80] suggested that the positive direct faecal PCR results may indicate presence of infection while other researchers [22, 77, 81] found that in absence of a more specific test (culture or F57 PCR), positive PCR results may indicate presence of mycobacterial DNA only. Ct values are inversely related to the amount of MAP in the sample 72, 82] and may be indicative of the presence of high shedders on farms. Although we applied the Ct cut off threshold of < 45 cycles for the test (as recommended by the manufacturer), Prendergast et al. (2018) [26] also found a somewhat similar threshold of 43.67 for this kit when applied to a well-defined sample population. As PCR testing is also limited by its inability to distinguish between viable and non-viable MAP cells [82, 83], caution in the interpretation of the PCR results is advised.

In our study, the culture positive samples were also positive on PCR (moderate Kappa value). Both culture and PCR results suggested the presence of high or moderate shedders in two farms. As no information was available on the MAP infection status of the herds it was not possible to draw any definitive conclusions on the respective merits of both tests for screening herds using environmental samples. As inclusion of more environmental samples does not improve the faecal culture positive recovery, repeated sampling of environment is recommended [69]. Although MAP herd prevalence in dairy herds is higher than in the beef herds in Ireland [55], environmental testing in beef herds is considered to be a reliable screening method for MAP [84].

In addition, storage of some samples for up to 6 months prior to culture may have resulted in a reduction of viable MAP in faeces [85], and may have accounted for some of the differences between the culture and PCR results presented in this study. Bovine salmonellosis is also a common disease on some Irish farms routinely identified as a cause of abortion or neo natal mortality and other infections [86, 87]. In 2015 *S*. Dublin, for instance, accounted for a total of 4.8% of the total foetal abortions [88]. *S*. Typhimurium is associated with acute enteritis [89, 90] and can survive in multiple different environments for extensive periods of time. According to Andino & Hanning (2015) [91] the prevalence of *Salmonella* in farm environments has been documented to range from 10 to 25%. Strohmeyer et al. (2006), Weese et al. (2005) and Joffe & Schlesinger (2002) [92–94] reported presence of *Salmonella* in 5.9, 20 and 80% of samples respectively in commercially available raw meats used for canine and feline diets. Rodrigues et al. (2006) [95] also reported the *Salmonella* presence in 10.4% of soil samples on bovine dairy farms. Although *Salmonella* spp. were not isolated from environmental samples in this study it may be due to the small number of farms tested and with testing only undertaken once during the spring months this may have limited the chances of its detection.

Antibiotic treatments given in animal husbandry are similar to human medicine [96]. As the amount of antimicrobial agents used for therapeutic and non-therapeutic purposes in agriculture is used for humans in many parts of the world [97], it is increasingly being considered a global health issue; both from the animal health and welfare aspect and because of the development of antibiotic resistance in animal pathogens [98, 99]. In addition, animal manure is a major source of antimicrobial resistant bacteria entering the environment, especially the soil used as fertilizer on agricultural land in the UK [100]. Much of this will contain low levels of antibiotics or antibiotic metabolites/conjugates, and antimicrobial resistant bacteria.

Non-pathogenic, multiple-drug-resistant *E. coli* in the intestine is an important reservoir of resistance genes [101–103]. The bacterium is one of the group of seven species that the world health organisation (WHO) has highlighted as of key AMR concern and serves as a sentinel organism for antimicrobial resistance in different types of animals. Because it is a common enteric commensal, it can be a pathogen, and easily acquires resistance and therefore can act as a reservoir that can transfer resistance to other species/pathogens [104–108]. Intestinal *E. coli* of animal origin may also colonize the human intestine, at least temporarily [109]. Bolton et al. (2014) [110] noted that over 60% of *E. coli* directly isolated were fully susceptible to the antibiotics tested with resistance where found being mainly to older antibiotics such as oxytetracycline and sulphonamide. Other studies [111] have reported most strains of ***E. coli*** isolated from cattle, were resistant to ampicillin (64%), tetracycline (74%), streptomycin (60%) and sulphonamide (76%) with low occurrence (1%) of enrofloxacin resistance, and in a later study by [112], all the ***E. coli*** isolates from dairy calves and lambs showed multi-resistance to tetracycline, streptomycin and compound sulphonamides with less resistance to enrofloxacin.

The emergence and spread of extended spectrum β-lactamase (ESBL)-producing *E. coli* associated with cattle and other farm animals [113–115] is a growing concern as both ESBL and AmpC β-lactamases can confer resistance to third-generation cephalosporins, penicillins and monobactams. These distinct enzymes can be differentiated by different susceptibility patterns against β-lactam site-specific inhibitors such as clavulanic acid and their differences in activity against fourth-generation cephalosporins [113, 116–118]. In this present study, 17.2% (27/157) of samples cultured on MAC agar plates containing 1 mg L^− 1^ CTX were identified as presumptive ESBL AmpC β-lactamase producing *E. coli*. These 27 presumptive ESBL AmpC β-lactamase producing *E. coli* underwent susceptibility testing which permitted isolates to be assigned ESBL or AmpC categories on the basis of their resistance patterns i.e., nine isolates (5.7% of samples) were identified to be ESBL producing *E. coli*, three isolates (0.2%) were identified to be ESBL and AmpC producing *E. coli* and 15 (9.6%) were identified to be AmpC producing *E. coli*. This trend i.e., highest number of presumptive ESBL AmpC β-lactamase positive samples identified as AmpC positive and lowest numbers positive for both ESBL AmpC β-lactamase has been previously reported to be observed in pig intestinal contents collected at slaughter during 2015 in Ireland and this trend has also been reported to be observed in the EU [119]. Carbapenemase-producing *E. coli* were not observed in the present study and this has also been reported previously by O’Sullivan et al. (2016) [119] for pig caeca, pork and beef during 2015 and chicken caeca and meat during 2016 in Ireland. Significant amounts of antibiotics are used within the agriculture sector in Ireland and it is estimated that 88% of veterinary antimicrobials administered consist of formulations of older antibiotics, such as penicillin, tetracycline and aminoglycosides [120].

The World Health Organisation (WHO) has initiated a number of global efforts to tackle the AMR problem including the categorisation of antimicrobials used in human health as critically important [121]. In 2014, the EU introduced harmonized monitoring of AMR across selected bacteria isolated from food and animals in Member States [19]. A panel of 14 antimicrobials were selected for monitoring using the micro broth dilution method EUVSEC Sensititre and EUCAST thresholds for resistance with testing undertaken in National Reference Laboratories, including our laboratory. While the results of this study demonstrated the prevalence of resistance to two critically important antibiotics, it was reassuring to note that the fluoroquinolone resistance was caused by a chromosomal mutation and not plasmid mediated i.e. resistant to both ciprofloxacin and nalidixic acid [122]. It would have been of value to carry out further analysis of these organisms using whole genome sequencing (WGS) to characterise the ESBL-encoding genes and identify additional antimicrobial resistance genes. In addition WGS would have been of value to determine genetic relatedness of the organisms by multi locus sequence typing (MLST).

The total tonnage of veterinary antibiotics used in Ireland was 103.4 t in 2016, and the most commonly sold antimicrobials for animal used in Ireland were tetracyclines (39.9%), sulphonamides & trimethoprim (20.7%) and penicillins (20.4%) [123]. This shows that tetracyclines and penicillins continue to comprise a significant portion of veterinary antibiotics used in Ireland. Also included in this report [123] were sales of 3rd and 4th generation cephalosporins and showed that these sales have generally remained unchanged over the last 4 years.

## Conclusions

The results of this study highlight the potential value of monitoring the farm environment for Johne’s disease; a growing concern for dairy and beef producers in Ireland and sampling the farm environment may provide a convenient way to rapidly screen herds for the presence of MAP. While it was reassuring that no carbapenemase-producing *E. coli* was observed in this study the presence of ESBL AmpC β-lactamase in environmental samples highlights the importance of monitoring samples for both cephalosporins and carbapenem classes since the *E. coli* producing ESBLs allows them to become resistant to most of the beta lactam antimicrobials. Further investigation of the value of farm environmental monitoring for Johne’s disease and AMR is recommended.

## Data Availability

The datasets used and/or analysed during the current study are available from the corresponding author on reasonable request.
